# The stagnation of innovation in humanitarian cash assistance

**DOI:** 10.1186/s41018-023-00136-3

**Published:** 2023-03-17

**Authors:** Alex Monich, Pablo V. Holm-Nielsen, Emmanuel Raju

**Affiliations:** 1 Global Health Section and COPE-Copenhagen Center for Disaster Research, Department of Public Health, University of Copenhagen, Øster Farimagsgade 5, Building 22, 1014 Copenhagen, Denmark; 2 Unit for Environmental Sciences and Management, African Centre for Disaster Studies, North-West University, Potchefstroom, South Africa

**Keywords:** Humanitarian organizations, Cash and voucher assistance (CVA), Innovation, Humanitarian sector

## Abstract

Cash and voucher assistance (CVA) has been gaining traction among humanitarian organizations as the preferred aid modality in disaster relief and complex emergencies. While the advantages of cash are well documented, the ongoing digitalization of cash and the emergence of innovative financial instruments can be associated with new operational challenges and a stagnation in innovation.

This paper reflects on the changing environment in CVA as a result of technological breakthroughs in the global financial system. The concept of humanitarian innovation is introduced to differentiate it from a similar process in the private sector and to investigate factors, contributing to a slower pace of acceptance, a reluctant implementation, or a complete rejection of innovative approaches in the humanitarian organization. The purpose of the study is to conceptualize the challenges of new technology adoption and scaling, as well as to analyze the direction and current stage of the diffusion of innovation in the humanitarian sphere.

Ten interviews with informants representing humanitarian agencies and the private sector were conducted to discuss their experiences with new CVA tools and perspectives on innovation in cash and voucher assistance. The results demonstrate that there is no uniform perception of innovation across the field, and the challenges of diffusion can be associated with several domains, including internal and external capacities, inherent characteristics of new financial technology (fintech), and a wider social, political, and regulatory context. According to the cyclical model of technological change, the innovative CVA fintech is currently at the ferment stage characterized by a high level of uncertainty and competition. The subsequent emergence of several dominant designs followed by incremental innovation is plausible in the future.

## Introduction

Cash and voucher assistance (CVA) is increasingly becoming the preferred modality for humanitarian intervention (CaLP 2020). Before the 2010s, CVA had accounted for a small share of the total aid volume—in 2006, it was less than 1% of the total humanitarian spending (Barder et al. 2015). However, the volume of CVA and its share in humanitarian assistance programmes have been increasing steadily during the past decade or more and reached 5.6 billion, or about 20% in 2019 (CaLP 2020). The increase in CVA volume is a reflection of many organizations adopting the approach as a fundamental mode of operation (Holm-Nielsen et al. 2022). According to experts’ estimates, CVA provides an opportunity to reach 18% more people with the same amount of funding, compared to in-kind contributions (Barder et al. 2015).

Recent technological advancements paved the way for further innovation in CVA and the potential introduction of new financial technology (fintech) tools, including distributed ledger technology (DLT), or blockchain, for aid distribution, identity management, or parametric insurance (CaLP 2020). Innovation in CVA is fueled both by humanitarian aspirations to increase the efficiency of aid delivery by organizations operating with chronically underfunded budgets, and external pressures of the modern information era, in which money is getting increasingly digitized and decentralized (Bergara and Ponce 2017). Recent developments in global finance, such as further growth and recognition of electronic payments and cryptocurrencies, the rapid growth of the non-fungible token (NFT)^1^ market; and the implementation of blockchain-based solutions across various industries from shipping to insurance, prompt policymakers to start preparing for a likely, if not inevitable, widespread propagation of fintech in the humanitarian field. In 2020, the United Nations declared that further digitalization of financial services is essential for achieving the Sustainable Development Goals (UN Digital Financing Task Force 2020).

Several pilot projects with DLT integration were initiated by humanitarian actors across the globe. However, despite the high overall level of support among donors and a significant growth of the CVA sector, the scaling prospects of new fintech tools have been unclear, and practitioners have expressed lower interest in further development of novel CVA approaches since 2017 (CaLP 2020). The adoption of innovative tools has demonstrated underwhelming results compared to the exponential growth of the CVA segment in the total volume of humanitarian assistance. A significant number of pilot projects have not progressed past their initial stage, getting stuck in the “proof of concept” level and demonstrating resistance to scalability (Lee 2020). The implementing organizations may not be adequately equipped to develop and maintain fintech solutions. Hence, an important aspect of CVA innovation is private sector involvement, which may include both large international companies behind the new technology and traditional local businesses participating in the implementation.

The concept of innovation in the private sector has been thoroughly investigated and some studies have pointed out its basic differences from innovation in the humanitarian sector. However, the challenges and implications of innovative CVA tools in the humanitarian sector have not been fully understood.

The aim of this paper is to analyze the innovation absorption and diffusion capacity of the humanitarian sector and assess existing and potential challenges and themes that impact humanitarian actors’ decisions to support or reject the implementation of novel CVA tools. The contribution of the study is to conceptualize challenges in CVA innovation by humanitarian actors and to explore internal and external factors contributing to the current stagnation in humanitarian fintech.

## Theoretical framework

### Innovation in the private sector

Innovation is defined as “the creative process whereby new or improved ideas are successfully developed and applied to produce outcomes that are practical and of value” (Taylor 2017). The phenomenon of innovation, primarily in the private sector, has been studied for many decades—the diffusion of innovation theory was first proposed by Everett Rogers in the 1960s (Rogers 1995). The theory describes how new ideas gradually gain popularity and become mainstream under the impact of three sets of variables, including inherent characteristics of adopters, pros and cons of innovation itself and a broader social and political context (Dearing and Cox 2018). Innovators can be represented by an organization if the diffusion is a system-wide effort across the organization or a selected individual within an organization who may face a certain degree of institutional resistance while recruiting early adopters within their own organization. The majority of innovations fail to diffuse and do not peak past the early adoption stage.

Innovative approaches and tools normally fall under one of two broad categories, known as either “process” or “product” innovation (Betts and Bloom 2014). As suggested by the taxonomy, product innovation is focused on significant improvements of an existing product or tool, or the development of a new tool, while process innovation emphasizes enhancements in methodology and program design.

With regards to the technological aspect of innovation, Anderson and Tushman (1990) proposed a cyclical model of technological change that describes the evolution of technology through a series of cycles, in which the introduction of brand-new products and services (technological discontinuity) opens a period of ferment, or fierce competition between different products that leads to the emergence of a dominant design or designs, while the rest of the products fail to diffuse. The emergence of the dominant design is followed by the era of incremental change (sustained innovation), where the existing design is gradually improved until the next discontinuity and the introduction of a new generation of products or services. Innovations at the beginning of the era of ferment are “crude and experimental” and the selection of a dominant design is largely a process of trial and error by implementing organizations (Anderson and Tushman 1990).

### Innovation in the humanitarian sector

Innovation in the humanitarian sector faces a unique set of challenges and does not necessarily have the same scope or rhythm as the similar process in the for-profit sector. Innovation in the humanitarian sector is presented as “an iterative process of implementing an idea” (UNHCR 2019). The concept of innovation is not exclusively associated with technology. UNHCR defines the following four dimensions of innovation: application of technology, application of innovative technology, innovative application of existing technology, and innovative application of innovative technology. The third is defined as the preferred type of innovation (UNHCR 2019).

Betts and Bloom outline several peculiarities of humanitarian innovation (Betts and Bloom 2014)—among other things, it is not driven by profit but rather based on humanitarian principles; there are inherent ethical concerns due to the precarious circumstances of affected people who should not be treated as untapped consumers; aversion to risk may discourage innovation; innovation is often driven by outsiders (including the private sector).

While the trial-and-error approach is acceptable with new products in the private sector, humanitarian principles and ethical considerations suggest that humanitarian innovation should be more risk-averse and participants are to be identified based on values rather than profit-seeking considerations (Batali et al. 2019). The do-no-harm principle is therefore crucial to the application of innovation in the humanitarian sector (Sandvik et al. 2017). Furthermore, aid recipients in difficult circumstances should not be equaled to untapped consumers in a new market and humanitarian aid is not profit-oriented (Betts and Bloom 2014), which is why a partnership with the private sector may present a challenge that has to be addressed before the implementation phase.

Christensen argues that it is the market-creating innovation that is the biggest contributor to development and poverty eradication (Christensen et al. 2018). However, in humanitarian studies literature, the stance on innovation is less radical since humanitarians by definition are more people-oriented and do not consider market related concerns very often as part of their work e (Lee 2020).

Innovation has not been unanimously embraced by humanitarian practitioners and researchers, with calls for further academic scrutiny of “utopian expectations” (Scott-Smith 2016; Sandvik 2017). The concept of innovation has been criticized due to its presumed tech-centeredness and an inclination to emphasize the novelty factor before the needs of potential beneficiaries (Scott-Smith 2016). Even with innovation proponents, the period of heightened enthusiasm sometimes was followed by disillusionment and rethinking of priorities, when innovation labs were declared dead (UNHCR Innovation Service 2018).

### Innovation in CVA

An important aspect of social and humanitarian innovation describes a “systems change” component, which refers to rethinking and redesigning existing social and economic systems through innovation (Papi-Thornton and Cubista 2019). Certain fintech components such as decentralized finance may be uniquely positioned to contribute to systems change along the lines of localization, sustainability and community ownership (Buterin and Weyl 2018). CaLP in its Outlook to 2030 suggests that further development of financial assistance will be a major contributing factor to the evolution of the entire humanitarian ecosystem over the next decade (IARAN and CaLP 2019).

Concrete examples of CVA innovation in the humanitarian sector have been analyzed elsewhere. Some of these examples include the World Food Program (WFP) Building Blocks in refugee camps in Jordan (Evans 2019), Unblocked Cash by Oxfam/Sempo in Vanuatu (Rust 2019), Community Inclusion Currency (CIC) by Grassroots Economics in Kenya (Bornstein 2019), DLT-based initiatives such as Sikka in Nepal (Rust 2019) and different varieties of charity coins (Farooq et al. 2020), and other initiatives like direct digital person-to-person donations (Gebken et al. 2021). Commercial tech giants have also expressed interest in the field. For instance, the Facebook-backed Diem (formerly Libra) Association includes several large humanitarian actors, pooling their efforts to develop a decentralized currency (CaLP 2020). The benefits and results of these programs have been shared by the implementers, based on two important aspects. One is that they have often not passed the proof-of-concept stage, showing that innovative CVA tools have not had the same level of growth as CVA programming in general, nor as similar fintech tools in the private sector. The second is that they all rely on a partnership with the private sector. There is however a knowledge gap in the way the perception of innovation and the implementing environment affect these aspects of CVA.

As financial instruments and technology become increasingly more sophisticated and the private sector gets actively involved, the privatization of the humanitarian sector has been argued to be challenging (Hotho and Girschik 2019). Private sector participation is crucial in aid delivery in general, but even more critical in innovative CVA, where financial service providers, software developers and platforms, mobile network operators and other actors will play an increasingly important role in facilitating transfers of both private remittances and institutional aid from humanitarian organizations (IARAN and CaLP 2019).

## Methodology

This study is based on qualitative research, based on a literature review on humanitarian innovation and 10 semi-structured interviews with practitioners from different international organizations either developing or implementing innovative tools. This method allowed for collecting open-ended data to explore the respondents’ thoughts on the topic allowing them to expand on their particular expertise and provide valuable insights (DeJonckheere and Vaughn 2019).

The literature review focused on theoretical foundations and the diffusion of innovation in humanitarian settings as well as characteristics of emerging CVA tools and the perception of innovation by humanitarian actors. The desk-based review of academic literature provided initial themes for the interview guide, while reports by associations such as Cash Learning Partnership (CaLP) helped to select innovative programs for further inquiry (IARAN and CaLP 2019; CaLP 2020).

Respondents included seven respondents working in humanitarian agencies and three representatives from the private sector focused on the impact of innovation and new fintech in CVA. Interview participants were selected through purposive sampling based on their practical experience with innovative CVA tools (Naderifar et al. 2017). The respondents were hence identified based on their experience with the phenomenon under study (Holzhauser 2008). Two of the humanitarians represented large organizations at the global level, while five others were working in the field. Private sector participants were representing tech companies and/or consultants offering specialized fintech solutions to humanitarian actors.

The semi-structured interview guide (Dearnley 2005) was informed by elements and potential challenges described in innovation studies and the cyclical model of technological change, such as familiarity and resistance to innovation, absorption capacity and stages in the diffusion of innovation. The thematic analysis was conducted under three categories based on the sets of variables that determine the diffusion of innovation described above: the inherent characteristics of adopters, challenges and opportunities of innovation per se, and a broader social and political context (Dearing and Cox 2018). Interview questions referred to interviewees’ personal experience with the respective CVA programs as well as their general opinions on innovation in the humanitarian sphere. Respondents were asked to provide their input on the role played by their own organizations, the private sector and government regulators in the successful implementation of innovative CVA programs.

Respondents’ identities were anonymized and marked P1-P10 in the interview notes—several participants specifically requested to stay anonymous and thus all interviewees’ identities were anonymized. P1–P2 are CVA experts working at headquarters of larger organizations, P3–P4 are private sector developers, and P5–P6 represent practitioners working in the field. Handwritten notes were taken during the interviews for preliminary identification of themes and concepts (Kvale 2011). The interviews were coded and cross-referenced in NVivo to identify and group themes and further structure the findings.

Since the paper focuses on practitioners’ perspectives, the comprehensive analysis of challenges and opportunities, associated with specific CVA tools and their impact on communities is beyond the scope of the study. Innovation in CVA is characterized by a rapidly changing landscape, and financial technology applications in humanitarian settings are predominantly new instruments with fluid features.

## Findings

Interview findings are grouped into three categories: particular characteristics of innovation in the humanitarian sector; challenges and opportunities of innovation in CVA; and characteristics of the enabling environment where CVA takes place.

### Innovation in the humanitarian sector

#### Perception of innovation and institutional resistance

Respondents showed a plurality of opinions on what innovation entails and how it should be implemented in the humanitarian sector. Their views varied from a slow and gradual introduction of selected new tools to a complete overhaul of the existing channels of financing and aid distribution, or systems change. The majority of respondents focused on the technological aspects of innovation and the broader implementation of new digital tools. Half of the respondents also emphasized the “innovative use of existing technology” and innovations in the policy space that are sometimes overlooked. One example that was often mentioned was new regulatory provisions for digital IDs.

The respondents reflected that the understanding of the importance and inevitability of innovation has been growing with humanitarian actors and the donor community. The COVID-19 pandemic provided an additional impetus to find new ways of distributing cash assistance. Nevertheless, all respondents emphasized that innovation must be context-specific. Specifically, two experts working in the field (P6 and P7) concluded further, that in certain contexts new fintech may be completely inappropriate as it imposes an additional burden on vulnerable individuals.

Respondents generally rejected the idea that technology-fueled innovation leads to an unnecessary degree of control by external actors. One reason is that humanitarian respondents believe that new tools can be owned and operated by communities themselves—localization is desirable and encouraged*.*

The respondents had different opinions on the prevalence of institutional resistance to change. In order to achieve a successful innovation process, the importance of organizational change for fostering innovation was explicitly mentioned by practitioners. One of the first steps would be the creation of a safe space for innovators. A few interviewees reflected that they initially struggled to generate enough support and enthusiasm within their own organizations for introduced innovation. In many cases, it was explained by the lack of knowledge and previous familiarity with technology. For instance, the prevailing myths and misconceptions surrounding blockchain, and the risky and volatile nature of cryptocurrencies.

Many participants explicitly mentioned the humanitarian-development nexus and the inherent contradiction between what was called “the disaster mentality” focusing predominantly on emergency response with short-term deployments, and long-term development objectives. With regards to CVA, it creates a disconnect between short-term cash injections in disaster relief, budget cycles, and certain innovative approaches that may require a longer time for rollout, onboarding and implementation.

The prevalence of the so-called “disaster mindset” was blamed for poor knowledge dissemination in the community. This mindset was described as practitioners flying in immediately after a disaster and for a short period of time and then leaving with no substantial knowledge for institutional memory*.*

#### Private sector involvement and cooperation

The role of the private sector, private-public partnerships, and the role of the government as a regulator/actor became crucial topics during the interviews. While the importance of partnering with the private sector for innovative solutions is recognized unanimously, there are diverging opinions on how this partnership should be shaped.

A humanitarian respondent from a global organization (P1) suggested that humanitarians should learn from the private sector and borrow their best practices, such as using the same instruments that financial institutions and countries use, and digital tools that have already been used and established in the private sector. At the same time, humanitarian respondents showed concerns about funds being channeled to the private sector, primarily large international corporations, instead of developing internal capacities.

The lack of standardization was emphasized as a serious challenge by two private sector participants (P3 and P4), who pointed to current fragmentation as one of the barriers to scaling existing innovation initiatives. However, there is an opportunity to find a common denominator as, according to the participant, the vast majority of humanitarian operations follow the same cycle and there is an opportunity to develop universal tools with some added flexibility. Furthermore, this lack of standardization (technical or otherwise), was identified as a barrier for further investment in the development of purpose-built solutions for the humanitarian sector. Standardization could help alleviate certain concerns related to partnerships with the private sector such as vendor lock-in^2^.

One respondent with field experience in conflict settings (P8) insisted on the integration of CVA into existing government programs and the national development agenda, suggesting that the government with its infrastructure and tools is best suited for delivery and there is no need to duplicate unless new tools and approaches fit into the existing framework.

### Innovation in CVA: challenges and opportunities

#### The perception of innovation in CVA

All respondents agreed that innovation of CVA will rely on the development of fintech. Three informants with different backgrounds (P1, P4 and P5) emphasized that fintech is neither good nor bad, it is neutral and can only be assessed as a component of cash-based programming: “The end goal is not technology. Giving out a digital ID does not ensure that you are now resilient” (P5). The do-no-harm principle was referred to by many respondents when discussing the implementation of technological innovation regarding CVA. Furthermore, most respondents pointed out that there is no need for innovation for the sake of innovation, the programming is shaped by humanitarian principles and is demand-driven; the end goal is not to be defined by technology but rather by what will have the biggest impact in terms of making the community more resilient to future disasters.

Respondents pointed out that innovation within CVA can trigger a transformation of the entire humanitarian ecosystem, from fundraising to long-term community development*.* The transformation may eventually result in more predictable funding based on parametric triggers and market-based instruments with lower transaction costs.

According to some respondents, another aspect of innovation in CVA revolves around digital ID management and connectivity. Digital ID is not necessarily tied to CVA; however, in many scenarios, digital ID has become a prerequisite for inclusion into CVA programming either because refugee populations are blocked from receiving IDs or buying SIM cards or if their IDs are insufficient for opening bank accounts due to AML and KYC regulations^3^.

One private sector participant (P4) and one staff member from a humanitarian organization (P1) advocated the development of a *common humanitarian platform* based on open-source software: “We're building a digital platform that allows us to pivot quickly from scenario to scenario, and to be able to be effective” (P1). This platform would enable users to build their own custom solutions based on a publicly available secure infrastructure for CVA implementation. Another private sector representative (P3) noted that while the idea sounds appealing in theory, it may be hard to implement in practice because of the current fragmentation and multiple stakeholders in the sector.

At the same time, two field experts (P6 and P8) expressed doubt that generalized solutions may be feasible, given the multitude of contexts, scenarios, and operational modalities and suggested that the emergence of a common platform is more likely in digital ID and data management than specifically in CVA implementation. Shared databases are in any case perceived as less secure by many humanitarian organizations. Furthermore, a single database maintained by an organization could also develop into a monopoly, which has more negative ramifications than positive outcomes.

#### Familiarity, learning, and failure

All the respondents referred to a lack of familiarity with new tools and approaches and relevant competencies as key challenges in implementation. Respondents further explained that familiarity and skillsets or competencies can be defined at the individual user level (beneficiaries), organizational level (humanitarian actors and private partners) and regulatory level (government). At the user level, familiarity with technology supports initial onboarding, implementation and troubleshooting. At the organizational level, it helps to overcome institutional resistance and generate support among other team members, who resist the introduction of technology and considerably slow down the process. At the regulator level, which is normally occupied by a responsible government agency, familiarity ensures faster approval and regulatory support of innovative mechanisms.

Many participants specifically mentioned how blockchain for many stakeholders is still exclusively associated with cryptocurrencies, while the range of possible applications of DLT goes far beyond cryptocurrencies. In the absence of alternative infrastructure, blockchain can be used as a backbone even for fiat currency distribution via tokens.

Most respondents pointed out that training requirements and formats also depend on the degree of previous familiarity—whenever stakeholders face an innovation for the first time, they need to be engaged differently: “Whenever you present something for the first time, you need to explain a lot of things; you need to engage them differently, communicating in a much simplistic way” (P4). The humanitarian respondents thought that it is important not to learn only within one’s own organization. Several respondents emphasized the value of cross-learning between organizations and between countries and contexts. For example, it was mentioned that governments seem to be more receptive to projects coming from other countries facing similar challenges.

Many respondents linked trust to familiarity, explaining that it is natural for people and communities to trust situations and organizations they have been previously exposed to and are familiar with. Respondents explained that trust can be considered at different levels, from beneficiaries to donors. Two participants who worked in countries with unstable financial systems (P7 and P9) suggested that in a situation, where official financial institutions cannot be trusted—for instance, when banks go bankrupt regularly and the deposits are not guaranteed—innovative financial technology may offer a viable and more trustworthy solution if properly implemented.

The importance of failure and its problematic exclusion from case studies was emphasized by two experts (P2 and P10), who pointed to a selection bias in industry reporting where only relatively successful pilot projects are reviewed outside of the implementing organizations: “Development and humanitarian actors need to understand that documenting innovation does include acknowledging failure” (P2). The omission of failed projects from published case studies jeopardizes cross-learning and keeps certain challenges hidden and under-analyzed. Considering that most innovations fail to diffuse, the exclusion of failures cuts off a major data source from the analysis. Reasons mentioned for the lack of reporting of failure were the self-perception of organizations and the fear that a damaged reputation may have on future funding. Creating a learning culture, which focuses on the analysis and documentation of successes and failures alike, was identified as an important step in organizational change and the successful implementation of innovation.

### Implementation environment

#### Government and regulatory framework

The role of the government and the importance of regulation was emphasized by all respondents, although there are different opinions on the degree of involvement since the government can be both an actor and a regulator in CVA. One participant working in the field (P8) advocated integrating CVA into the existing government transfer scheme, arguing that humanitarian organizations underutilize existing infrastructure for fear of making themselves redundant. However, all respondents pointed out that changing government regulations is a lengthy process that remains one of the most significant challenges.

Similar to humanitarian organizations, national regulatory frameworks were criticized by participants for lagging behind the latest technological developments, which may cause friction between financial regulators and fintech innovators if they are perceived as parallel currency issuers: “Who gave you the right to print money in our country? You know, there's a lot of that sort of vibe around it as well” (P10). Some governments have instituted outright bans on cryptocurrencies but allow the use of blockchain technology as an infrastructure for supply-chain management and other non-monetary applications. In one case, the use of external cryptocurrencies was not allowed but the innovation lab was granted permission to mine their own tokens for use in CVA, and the NGO is now looking at exporting this format to other countries*.* The absence of clear regulations means that humanitarian actors often operate in a grey area.

One participant (P2) stated that the government can also be quite enthusiastic about innovation, especially if the competent authorities are somewhat familiar with the project, or if it has already been implemented in a similar context (e.g., other countries in the region). Government support is instrumental in digital ID projects—for example, it is the primary responsibility of governments to manage the identities of hosted populations but there are currently persistent gaps with regards to refugee ID management. In conflict scenarios such as Syria for example, the government can restrict access to certain areas and population segments.

#### Localization and CVA innovation

One aspect that was repeatedly mentioned concerning CVA innovation was localization. The majority of interviewees from humanitarian organizations believed that innovative tools are more likely to be successful if they are developed and implemented by local organizations. Even when an external solution is used, certain components of a tool or its implementation can and should be localized.

Partnership with local for-profit companies was seen as less challenging compared to large transnational corporations. However, it was pointed out that certain provisions of NGO procurement policies lead to the prioritization of more established vendors who meet the required criteria, which is problematic for inclusive community development.

Respondents mentioned that localization and knowledge dissemination is a lengthy process. Hence the challenge of the above-mentioned “disaster mentality” leading to high staff turnover and short-term cash injections can be detrimental. Physical and digital connectivity is crucial for the community’s ability to absorb knowledge and develop innovations locally. Local actors are more likely to reach a better level of understanding with the national government.

Respondents empathized that CVA innovation can foster localization if implemented the right way but can also lead to an impediment to localization if technology is not shared with local partners or if knowledge and resources are not available locally.

#### Digital divide and the future of CVA

All participants affirmed the need for a context-driven and demand-driven nature of humanitarian innovation. This would mean that CVA programming should be custom-made to address the needs of a selected community, and that specific CVA tools and modalities selected should maximize a positive and long-lasting impact on the community.

Even though digital literacy has been growing rapidly in developing countries (Ameen and Gorman 2009), the digital divide still poses a serious challenge. One of the respondents with field experience (P5) stated that digital inclusion is one of the objectives of humanitarian work in general: “As you're looking at more of these digital IDs or peer-to-peer payments or cryptocurrencies, you must ensure that the rights of the individuals are not being impeded. Making sure that you are not excluding certain groups, because they are not able to, to attain, or get access to that digital or technological aspects” (P5). Therefore, it was argued that improving connectivity and infrastructure should be part of CVA programming and wider development initiatives.

The interviewees expressed unanimously that the humanitarian field is changing rapidly. Innovation, therefore, becomes a driver and a product of demand-driven organizational reforms at the micro level and is fostered by the changing landscape at the macro level. People’s understanding of the concept of money is gradually changing, so the awareness about alternative payment methods will be growing and the demand structure will be changing as well. The evolution of innovation in the humanitarian sector will therefore potentially be dictated by the evolution of fintech, which is in disagreement with the current stagnation in humanitarian fintech innovation.

There were other related aspects of the innovation of CVA that are likely to influence the humanitarian sector according to our respondents:

Half of the respondents were hoping to have a more predictable funding mechanism in the future, and two respondents with different backgrounds (P1 and P10) outlined a system change scenario, in which new CVA tools will be integrated into a comprehensive funding system with market-based fundraising elements.

Also, the humanitarian respondents believe that the gap between the humanitarian and the development side will be bridged, and a new funding mechanism will provide a degree of flexibility without time limits imposed by external actors. This will effectively blur the line between CVA and UBI.

Finally, the COVID-19 pandemic exposed a significant number of people in both developed and developing countries to various cash assistance programs and is likely to become an additional powerful contributor to innovation in CVA, given the large number of emergency assistance programmes and the restrictions imposed by the pandemic.

## Discussion

Our findings indicate that innovation in the humanitarian sector does not necessarily follow the same diffusion principles that were described in the private sector (Rogers 1995) and possesses certain characteristics that are unique to humanitarian innovation (Betts and Bloom 2014). Further, we discuss the perception and effectiveness of innovation in the humanitarian sector and analyze its current state and the future of the innovative process with regard to new CVA tools. The challenges mentioned below are associated with the role of the private sector, the regulatory environment and institutional rigidity of the humanitarian sector.

### Innovation in the humanitarian sector

The interviews demonstrate that humanitarian actors subscribe to multiple definitions of innovation in CVA and the ways it should be implemented. The transition from in-kind assistance to cash-based programming is viewed as an example of *process* innovation, whereas the introduction of novel CVA tools (e.g., blockchain-based transfers or identity management solutions) can represent both process and *product* innovation as a combination of the two domains, which may eventually result in paradigm change reflecting a complete replacement of in-kind assistance with CVA (Ramalingam et al. 2009).

It can be argued that innovation in the humanitarian sector is slower and less efficient compared to the private sector due to inherent characteristics of the humanitarian field. Humanitarian innovation does not always match the conventional understanding of innovation in the private sector, nor does it always follow the same diffusion pattern. On the one hand, humanitarian organizations are willing to go beyond strictly technological solutions and include unconventional applications of traditional tools and community-based informal systems; but on the other hand, they tend to focus more on either sustained or efficiency innovation since ethical considerations and the nature of humanitarian work prevent from initializing more radical market-creating or disruptive innovations.

As our findings show, the slower pace and the emphasis on sustained, less radical innovation is caused by several factors. Although innovation is demand-driven both in the open market and in humanitarian scenarios, basic ethical considerations postulate that disaster affected populations are different from untapped consumers and market creation is hardly appropriate in humanitarian settings. In addition, since CVA is highly context-specific, it often requires custom-built tools as opposed to truly global products.

According to our respondents, this kind of fragmentation and the lack of common understanding of digitalization among strategists and practitioners is a significant barrier to scaling—if the definitions and objectives of innovation do not match, neither will the metrics for success and failure. Better understanding and harmonization of concepts can be facilitated by sector associations like CaLP and further standardization of technology and procedures, provided that humanitarian organizations are flexible enough to absorb and process external knowledge.

### Era of ferment

Our findings demonstrate that the current landscape of CVA innovation is characterized by fragmentation and a high level of uncertainty. According to the cyclical model of technological change, the present phase of fintech in CVA matches the ferment era of the innovation cycle (Anderson and Tushman 1990), where multiple competing tools are piloted by different organizations and the dominant design may slowly emerge. The majority of innovations fail to diffuse; hence failures are common. The analysis of failures at this stage could provide valuable data for the next ferment cycle. However, our research shows that implementation failure is not shared often with the humanitarian community, which hinders true knowledge sharing. Factors for not sharing failed attempts at innovation implementation include the perception and reputation of the organizations and fear of influence it might have on future funding.

Only after a dominant design or designs emerge, the innovation cycle will reach maturity (Anderson and Tushman 1990). At the same time, it can be argued that CVA in general has already established itself as a dominant design regarding it as an outcome of process innovation in an earlier cycle. Thus, fintech represents a new cycle within a cycle as a result of technological discontinuity that gave rise to new digital tools. However, the humanitarian innovation based on fintech is currently characterized by stagnation.

In the best-case scenario, the emergence of dominant designs and potentially global platforms in CVA may foster a period of collaboration, with multiple organizations focusing on the improvement of the selected tools (IARAN and CaLP 2019). However, it must be noted that collaboration is when organizations truly work with one another beyond mere information sharing (Raju and Becker 2013). It is yet unclear whether these designs will emerge as a result of a deliberate coordinated and collaborated efforts or replicate organically from successful pilot projects. The era of ferment can produce several flexible context-specific tools and shared platforms with industry-wide standards for further improvement during the subsequent era of incremental change.

In its Outlook for 2030, CaLP proposes four basic scenarios of the sector evolution: control; chaos; emergence; and synergy; with the latter scenario being the most positive one (IARAN and CaLP 2019). According to CaLP, the synergy is unlikely to happen before 2025; yet many study respondents are quite optimistic about their scaling perspectives, hence the emergence and replication of dominant designs may happen even sooner either on a regional or global level. The transformation of the global financial market and the growing role of fintech will change the CVA landscape and the humanitarian organizations that administer aid delivery. The complexity of new fintech tools means that partnerships with the private sector are not just desirable but also unavoidable.

### Private sector—friend or foe?

Our findings show that partners from the private sector have to be brought in to offer unique expertise, bridge knowledge and capacity gaps, explore new opportunities and find efficiencies. However, these partnerships can introduce new challenges; there is a plurality of views on the role and underlying motivations of the private sector, with a notable degree of discomfort caused by the potential privatization of the humanitarian sphere. Maximizing profit may not always be consistent with humanitarian principles (Betts and Bloom 2014); hence, it is important to find areas of the most optimal overlap and alignment of objectives. As mentioned above, an ethical approach dictates that there is a difference between market-creating for-profit innovation and humanitarian settings, in which affected people should not be treated as potential new customers. Along with privacy and security concerns, collecting large volumes of sensitive personal data also raises an important question if humanitarian organizations will become ‘data brokers’ in the digitization process (Lemberg-Pedersen and Haioty 2020).

The importance of collaboration with and learning from the private sector is emphasized in major industry reports (CaLP 2020), yet the format and the boundaries of this partnership are not properly established. In fact, given the variety of contexts and stakeholders involved, it is unlikely that such standard rules of engagement can be introduced at all. Further, an avenue to be examined is the clash of ideologies and values in the different sectors. The criticism of privatization usually focuses on larger corporations, even though the collaboration framework between a humanitarian organization and the private sector can engage local small and medium-sized enterprises. This kind of collaboration is crucial for localization. There seems to be a tendency to view local businesses and organizations in a more positive light compared to transnational corporations.

Localization is a popular concept in the humanitarian sector and was one of the workstreams under the Grand Bargain (WHS 2016). And although there has been some success with the localization-related commitments of the Grand Bargain over the last 5 years (Metcalfe-Hough et al. 2021), innovative approaches are often characterized as exogenous, and the bottom-up innovation is not given enough credit. Our respondents reflected that a meaningful engagement of local businesses can contribute to bridging this gap. Both with national and international companies, data privacy and data protection are sensitive matters—with the inclusion of additional players into the ecosystem, the likelihood of a sensitive data leak increases.

Although some participants from the humanitarian sector expressed their indignation with the level of private sector involvement and the amounts paid to private software developers and other contractors, a complete detachment from the private sector does not seem plausible. Given the sophistication of new fintech and the competencies required for in-house development, very few organizations can have sufficient funds and human resources to spend on self-produced tools. In addition to that, our data show that the private sector would favor a larger standardization of the humanitarian sector, in order to develop effective technical tools and scaling up proof-of-concept initiatives.

### Other challenges

Our findings suggest that institutional resistance to innovation and the prevalence of the so-called “disaster mindset”, meaning a propensity to apply short-term patchwork solutions to humanitarian crises, can highlight the need for organizational change. The institutional resistance can manifest in different shapes and forms—from an outright and permanent ban on certain kinds of innovative approaches in CVA to a subtle underappreciation or lack of investment into approaches that may be considered too radical and/or not fitting into the established organizational format. The rigidity and conservatism of organizational structures in the humanitarian sector and actors’ inability to quickly adapt to a changing environment has been a topic for discussion for a long time, some scholars call the system completely broken in the absence of a joint political effort for transformative action (Spiegel 2017).

Although this kind of pessimism was not fully shared by study participants, the issue of organizational change and flexibility to support humanitarian innovation takes a central place in the discussion. The ability of humanitarian organizations to change under the influence of external push and pull factors was described in the literature more than a decade ago (Clarke and Ramalingam 2008), although the effectiveness and the extent of such changes are up for discussion. Following our findings, the agent of change and the driver of innovation can be either internal, i.e., an influential member of the organization, who supports and promotes innovation and the required changes in the organizational fabric; or external, when changes become inevitable due to dramatic events in the operational environment. As a technological discontinuity, blockchain generated new financial and supply chain management tools, while the covid-19 pandemic and lockdowns resulted in a skyrocketing demand for innovative aid distribution tools that could be managed remotely. These dramatic changes will undoubtedly affect even the most conservative humanitarian organizations that would otherwise prefer to continue with their business-as-usual approach.

When it comes to the regulatory framework, our findings indicate that the government’s willingness to cooperate and accommodate is a key factor contributing to the successful implementation of innovative CVA programs. In the humanitarian field, governments act in a dual capacity concerning innovation—both as a regulator and also as one of the main providers of assistance. In its role as a regulator, the government to a large extent shapes the context in which other actors must operate. Governments across the world employ drastically different approaches to blockchain, ID digitalization, and legal requirements. Therefore, cross-border scaling and deployment can be impeded by non-uniform regulatory environments and a lack of standardization in different countries and regions. If the government is supportive of innovation and the humanitarian programming fits into the national political and development agenda, it becomes a strong contributor to the successful diffusion of innovative tools and approaches.

## Conclusion

Our research shows that the humanitarian sector welcomes innovation but is not necessarily well-equipped to implement it. The plurality of views and opinions on the appropriateness of innovation in various humanitarian scenarios further contributes to stagnation of fintech in CVA. On top of that, humanitarian actors often have to count on the vision and values of their private sector partners. Given the complexity and sophistication of new technology, private sector involvement in innovative CVA will necessarily continue to grow in the foreseeable future. It is important to develop a meaningful collaboration framework early in the development cycle. Furthermore, national and supranational regulators can contribute to this assignment as well—for instance, with data protection rules.

The diffusion rate of innovative tools depends on whether stakeholders can reach a consensus on their vision for the future of CVA and the humanitarian sector in general. In a lot of ways, humanitarian innovation follows classical patterns with an initial institutional resistance, a high failure ratio and challenges caused by lack of familiarity. A slower pace of innovation diffusion and fintech development in the humanitarian sector is determined by the inherent characteristics of humanitarian actors and the necessity to maintain a delicate balance between the efficiency of the innovative cycle and the human-centeredness of needs-based programming. Organizational changes can be harder to implement in the humanitarian community, and in many cases, institutional reforms are required across the board—from governments to donors to implementing organizations. Another aspect that will facilitate innovation is creating a learning culture, which focuses on the analysis and documentation of successes and failures alike and concentrates on local infrastructure, resources and knowledge, thus aligning the paths between innovation and localization in CVA.

The innovation challenges are persistent but not insurmountable. The near future will demonstrate whether humanitarian innovators can push their pilot projects past the ferment stage and into a large-scale deployment. The ferment stage provides an opportunity to study successes and learn from failures. Due to the level of required expertise, the learning curve is likely to be steeper compared to CVA adoption over the last two decades but those who manage to advance are likely to determine the dominant toolset for the future.

## Data Availability

The data generated and analyzed during the current study is based on the conducted interviews. The data are not publicly available due to the respondents being assured that they would remain anonymous, as explained in the Methodology section.

## Footnotes

1 NFT is a unique unit of data stored on blockchain that can be sold or traded. It is often associated with digital art.

2 Vendor lock-in refers to the scenario when someone is forced to use a product or service regardless of its quality (often subpar) due to dependency and/or substantial switching costs.

3 Anti-Money Laundering and Know Your Client regulations specify verification procedures and documents required for opening bank accounts and other transactions.
